# Discovery of novel astrovirus and calicivirus identified in ruddy turnstones in Brazil

**DOI:** 10.1038/s41598-019-42110-3

**Published:** 2019-04-03

**Authors:** William Marciel de Souza, Marcílio Jorge Fumagalli, Jansen de Araujo, Tatiana Ometto, Sejal Modha, Luciano Matsumiya Thomazelli, Edison Luís Durigon, Pablo Ramiro Murcia, Luiz Tadeu Moraes Figueiredo

**Affiliations:** 1 Virology Research Center, School of Medicine of Ribeirão Preto of University of São Paulo, Ribeirão Preto, 14049-900 SP Brazil; 2 MRC-University of Glasgow Centre for Virus Research, Glasgow, G61 1QH Scotland United Kingdom; 3 Department of Genetics, Evolution and Bioagents, Institute of Biology, University of Campinas, 13083-862, Campinas, São Paulo, Brazil; 4 Institute of Biomedical Sciences, University of São Paulo, São Paulo, 05508-900 SP Brazil

## Abstract

Birds are the natural reservoir of viruses with zoonotic potential, as well as contributing to the evolution, emergence, and dissemination of novel viruses. In this study, we applied a high-throughput screening approach to identify the diversity of viruses in 118 samples of birds captured between October 2006 to October 2010 in the North and Northeast regions of Brazil. We found nearly complete genomes of novel species of astrovirus and calicivirus in cloacal swabs of ruddy turnstones (*Arenaria interpres*) collected in Coroa do Avião islet, Pernambuco State. These viruses are positive-sense single-stranded RNA with a genome of ~7 to 8 kb, and were designated as Ruddy turnstone astrovirus (RtAstV) and Ruddy turnstone calicivirus (RTCV), respectively. Phylogenetic analysis showed that RtAstV and RTCV grouped in a monophyletic clade with viruses identified from poultry samples (i.e., chicken, goose, and turkey), including viruses associated with acute nephritis in chickens. Attempts of viral propagation in monkey and chicken cell lines for both viruses were unsuccessful. Also, we found genomes related with viral families that infect invertebrates and plants, suggesting that they might be ingested in the birds’ diet. In sum, these findings shed new light on the diversity of viruses in migratory birds with the notable characterization of a novel astrovirus and calicivirus.

## Introduction

Astroviruses and caliciviruses are positive-sense single-stranded RNA with a genome of 6.8 to 8.3 kb^1,2^. These viruses spread primarily to vertebrates via the fecal-oral route and are associated with gastroenteritis worldwide^1–3^. Currently, the *Astroviridae* family is composed of 21 viral species, which are divided into two genera, the *Avastrovirus* genus that infects avian species, and *Mamastrovirus* genus that infects mammals including humans^4^. Avastroviruses are emerging pathogens that have been associated to cause diverse pathologies in birds, including enteritis, hepatitis, and nephritis, which have been associated with economic losses in the poultry industry (e.g., white chick disease)^5–8^. Additionally, mamastrovirus infections are characterized by gastroenteritis and in rare cases cause neurological syndromes and encephalitis^1,9–11^. Human astroviruses (HAstV) are recognized to cause childhood viral gastroenteritis worldwide, but it has only been associated with neurotropism in immunocompromised patients^3,12–14^.

The *Caliciviridae* family comprises seven viral species classified into five genera: *Lagovirus*, *Nebovirus*, *Norovirus*, *Sapovirus*, and *Vesivirus*^15^. Caliciviruses naturally infect a broad spectrum of vertebrates including humans, cows, pigs, cats, chickens, reptiles, dolphins, fish, and amphibians^16–25^. Human diseases due to calicivirus infections are mainly caused by Noroviruses (i.e., Norwalk virus), which are the second most common causative agents of viral gastroenteritis in the world^25^. Also, they have been associated with relevant veterinary diseases, such as respiratory illnesses in cats caused by feline calicivirus, and hemorrhagic fever in rabbits^18,26^. Currently, there are not suitable cell culture systems or animal models to study calicivirus pathogenesis, except some for noroviruses, but these are not easy to reproduce^27,28^.

Studies based on high-throughput sequencing (HTS) have expanded the known viral and host diversity of astroviruses and caliciviruses, providing insight on their transmission dynamics in nature^29–32^. In this study, we used HTS metagenomics to identify the viral diversity in birds captured in the North and Northeast regions of Brazil.

## Results

Analysis of fourteen metagenomic datasets derived from pools of cloacal swabs and blood samples generated a total of 7,974,180 to 29,350,054 paired-end reads with 72.08% to 88.60% of bases ≥Q30 with a base call accuracy of 99.90% (Table 1). First, we used MetaViC to remove non-viral sequences and remaining reads were *de novo* assembled. A total of 83 to 99.5% of reads were classified as eukaryote and bacteria, and unclassified reads were identified in 0.3 to 1% total reads in six pools (Supplementary Table 1). We identified 0.5 to 17% of contigs exhibiting similarities with viral genomes. Viral contigs with ≥750-bp in length were used in downstream analyses.

**Table 1:** Information of sample pools used in this study.

Pool	Specie	Sample	N^a^	Place	Date	Reads	Q30
1	*Gallus gallus*	Cloacal Swab	3	Breves, PA	30/10/2006	7,974,180	80.97
2	*Cairina monchata*	Cloacal Swab	12	Breves, PA	30/10/2006	17,494,780	80.84
3	*Cairina monchata*	Cloacal Swab	12	Breves, PA	30/10/2006	24,336,384	88.17
4	*Cairina monchata*	Cloacal Swab	17	Breves, PA	30/10/2006	18,749,660	88.02
40	*Arenaria interpres*	Cloacal Swab	15	Coroa do Avião, PE	06/10/2010	18,609,356	88.60
41	*Arenaria interpres*	Serum	14	Coroa do Avião, PE	06/10/2010	24,721,100	86.97
42	*Thalasseus sandvicensis*	Cloacal Swab	5	Coroa do Avião, PE	06/10/2010	19,600,110	88.37
43	*Thalasseus sandvicensis*	Serum	3	Coroa do Avião, PE	06/10/2010	20,752,238	87.41
44	*Arenaria interpres*	Cloacal Swab	4	Coroa do Avião, PE	10/2010	25,344,398	88.17
45	*Calidris pusilla*	Cloacal Swab	9	Coroa do Avião, PE	10/2010	22,012,904	86.94
46	*Calidris pusilla*	Cloacal Swab	9	Coroa do Avião, PE	22/09/2010	29,350,054	87.30
47	*Hilophilus amaurocephalus*	Cloacal Swab	4	São José do Egito, PE	22/09/2010	24,327,186	72.08
48	*Sarkesphorus cristapus*	Cloacal Swab	6	São José do Egito, PE	23/09/2010	22,988,194	74.32
49	*Coryphospingus pileatus*	Cloacal Swab	4	São José do Egito, PE	22/09/2010	17,662,972	86.88

N^a^: number of individual per pool.

### Genomic characterization of a novel astroviruses

**Figure 1:**
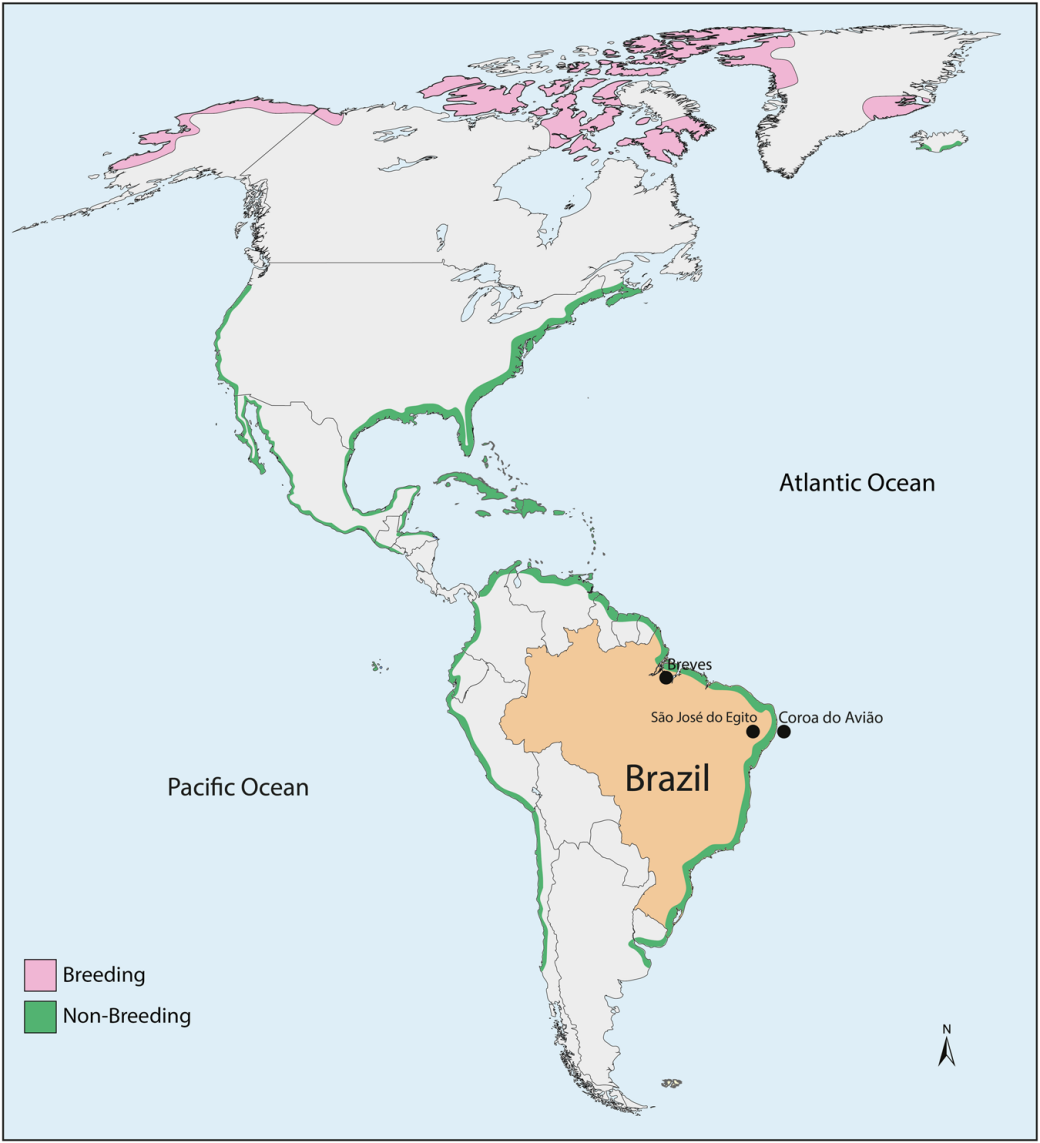
Geographic locations of sampling sites in Brazil. Sites are shown as black circles. Distribution of the Ruddy Turnstone in America, the breeding regions are shown at pink color and non-breeding regions are shown at green color. The map is a modified version of the original BirdLife International. 2016. *Arenaria interpres*. The IUCN Red List of Threatened Species 2016: (https://www.iucnredlist.org/species/22693336/86589171), licensed under a Creative Commons Attribution 4.0 Unported License (https://creativecommons.org/licenses/by/4.0/).

**Figure 2:**
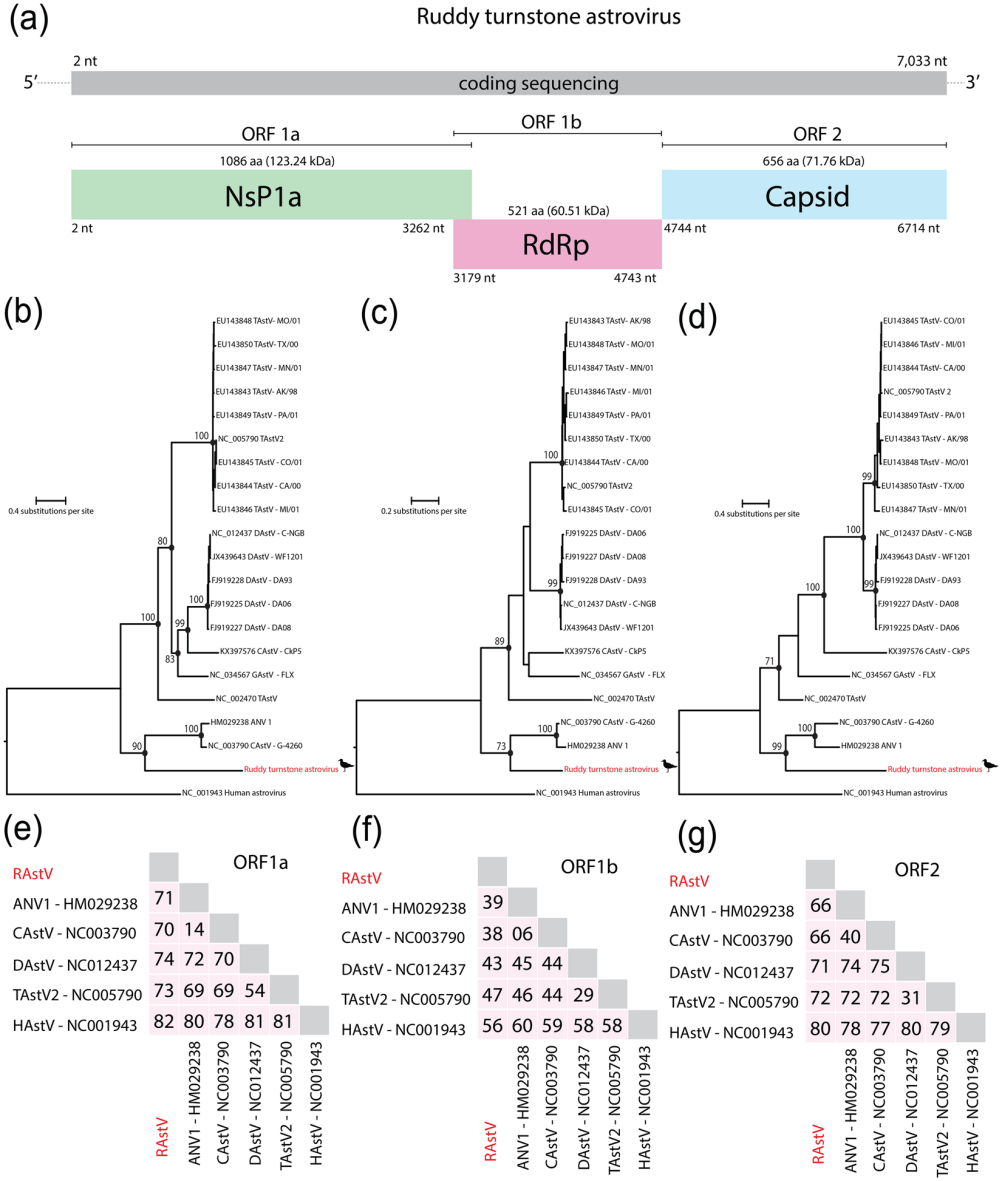
(**a**) Genome organization of the complete coding sequence of Ruddy turnstone astrovirus (Genbak acession number MK189093). (**b**) Maximum likelihood phylogenies of Ruddy turnstone astrovirus into *Avastroviruses* genus. Trees were inferred on amino acids alignments of ORF1a (**b**) and ORF1b (**c**) based on LG + I + G4 amino acids substitution model, and ORF2 (**d**) based on LG + F + G4 amino acids substitution model. Phylogenies are midpoint rooted. The scale bar indicates evolutionary distance in numbers of substitutions per amino acid site. Bootstrap values of 1,000 replicates are shown in principal nodes. Ruddy turnstone astrovirus sequence is shown in red. Amino acid divergences of Ruddy turnstone astrovirus and representative avastroviruses related of ORF1a protein (**e**), ORF1b protein (**f)** and ORF2 protein (**g**).

We identified a nearly complete genome of a novel astrovirus species in a pool of cloacal swabs (Pool 40 – Table 1) derived from ruddy turnstones (*Arenaria interpres*) collected in Coroa do Avião islet, Pernambuco State, Brazil (Fig. 1). This virus was tentatively designated as Ruddy turnstone astrovirus (RtAstV). RtAstV presents a typical genome organization of astroviruses, which consists of a single-stranded positive RNA of 7,033 nucleotides (nt) composed by three open reading frames (ORFs), named ORF1a, ORF1b, and ORF2 (Fig. 2a). The RtAstV genome was obtained by 56,329 reads with a median coverage of 3,006x (Supplementary Fig. 1). ORF1a is 3,261 nucleotides (nt) long and encoded the putative viral protease (NsP1a polyprotein), which is 1,086 amino acids (aa) long. ORF1b is 1,566 nt long, and encoded the putative RdRP protein, which is 521 aa long. We identified an overlapping region between ORF1a and ORF1b of 85 nt. ORF2 is 1,971 nt long and encodes a 656 aa long capsid precursor protein. Based on BLAST analysis, RtAstV shares 28 to 60% of amino acid identity with chicken astrovirus (GenBank No. NC_003790).

To determine the frequency of RtAstV within our pools, we screened all individual samples by RT-PCR^33^. RtAstV was detected in only one sample from the same pool in which RtAstV was identified using our HTS approach. Phylogenetic analysis based on amino acids sequences of ORF1a, ORF1b, and ORF2 revealed that RtAstV clusters in a monophyletic clade with strains of avastroviruses, which are associated with acute nephritis in chickens^8,34^ (Fig. 2b–d). No evidence of recombination was observed in RtAstV. Based on pairwise distance analysis, we identified that RtAstV shares with other avastroviruses 71 to 82% amino acids distance in nsP1a polyprotein (ORF1a), 39 to 56% amino acids distance in RNA-dependent RNA polymerase - RdRP (ORF1b), and 66 to 80% amino acids distance in precursor capsid protein (ORF2) (Fig. 2e–g).

We attempted to isolate RtAstV in two different cell lines: UMNSAH/DF1 (chicken) and Vero (African green monkey). To this end, homogenates from a single sample was inoculated in cell monolayers, and the supernatant of infected cells was serially passaged three times. Viral sequences were detected by RT-PCR at day seven post-infection in the first passage in both cell lines, but not in further passages. No cytopathic effect (CPE) was observed.

### Genomic characterization of a novel calicivirus

**Figure 3:**
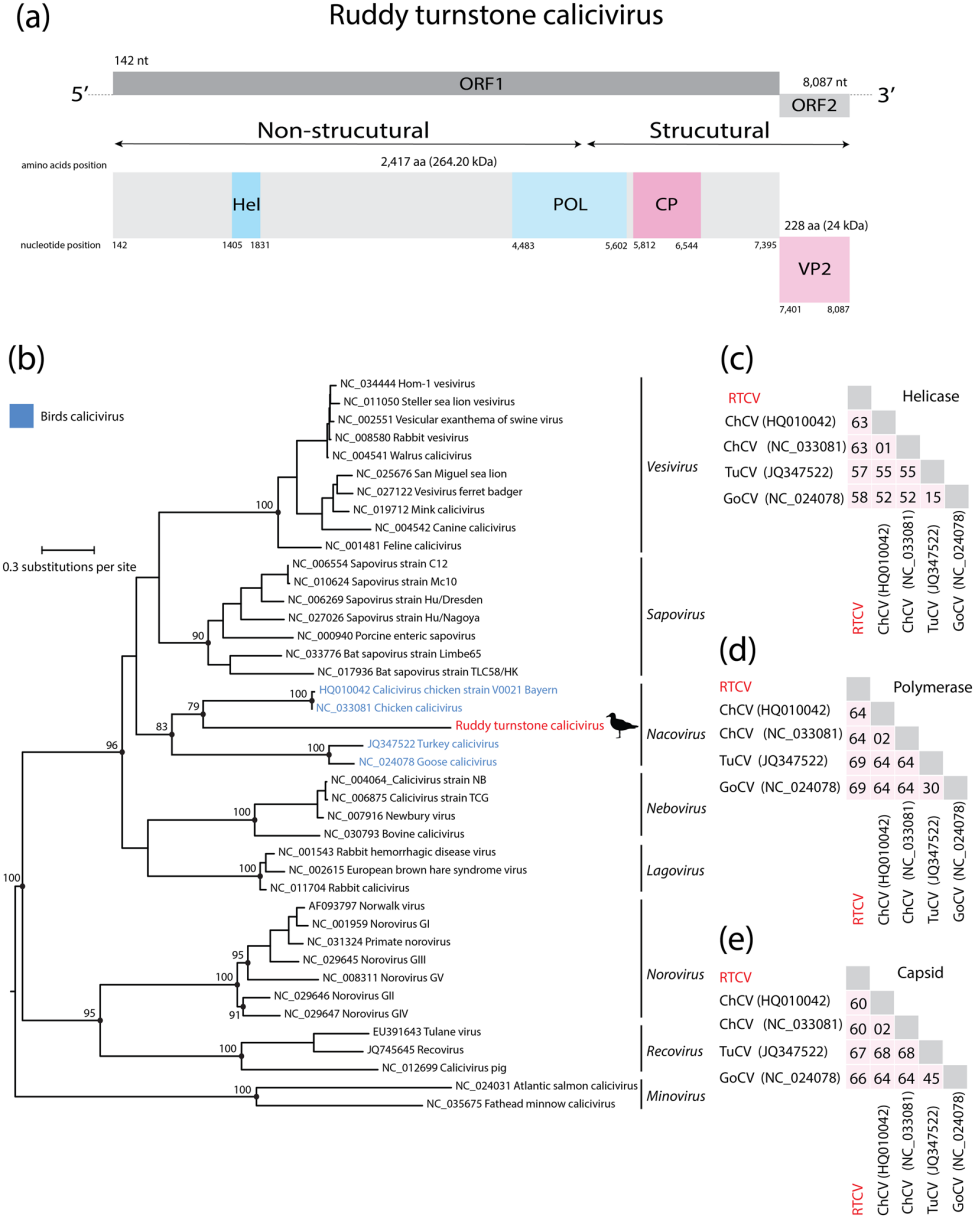
(**a**) Genome organization of the complete coding sequence of Ruddy turnstone calicivirus (Genbak acession number MK189094). (**b**) Maximum likelihood phylogeny of the *Caliciviridae* family. Trees were inferred using amino acids alignments of the polymerase domain based on LG + I + G4 amino acids substitution model. Phylogenies are midpoint rooted. Scale bar indicates evolutionary distance in numbers of substitutions per amino acid site. Bootstrap values of 1,000 replicates are shown in principal nodes. Ruddy turnstone calicivirus sequence is shown in red. Amino acid divergences of Ruddy turnstone calicivirus and representative caliciviruses related of helicase domain (**c**), polymerase domain (**d**) and capsid domain (**e**).

A nearly complete genome of a novel calicivirus was identified in a pool of cloacal swabs (Pool 44 – Table 1) derived from ruddy turnstones collected in Coroa do Avião islet, Pernambuco State, Brazil (Fig. 1). This virus was tentatively designated as Ruddy turnstone calicivirus (RTCV). RTCV has the typical genome organization of caliciviruses, with a single-stranded positive RNA of 8,127 nt, which encodes two proteins, the polyprotein (i.e., helicase, polymerase, and capsid) and the VP2 protein. The RTCV genome was obtained by 4,817 reads with a median coverage of 258x (Supplementary Fig. 2). The ORF1 polyprotein is 7,254 nt and encodes a 2,417 aa long polyprotein, which includes Helicase (Hel), Polymerase (Pol) and Capsid (CP) predicted domains. ORF2 is 687 nt long and encodes a 228 aa long viral protein 2 (VP2) (Fig. 3a). Based on BLAST analysis, the RTCV polyprotein is 28% identical to that of chicken Calicivirus (GenBank No. HQ010042). RT-PCR screening of all individual samples showed that only one sample was positive to RTCV (as expected, within the same pool in which we detected RTCV using our HTS approach).

We performed phylogenetic analysis using protein alignments of the polymerase domain that included the RCTV genome together with other 40 representative sequences of the *Caliciviridae*. RTCV clustered in a monophyletic clade with caliciviruses identified from poultry samples (i.e., chicken, goose, and turkey) (Fig. 3b). Based on amino acids p-distance analysis, RTCV shares 58 to 64% amino acids in helicase, polymerase and capsid domains with other caliciviruses previously described (Fig. 3c–e). We also attempted to isolate RTCV in UMNSAH/DF1 and Vero cells as described above. RTCV was detected by RT-PCR at seven days after inoculation in the first passage in UMNSAH/DF-1 cells only, but no virus was detected in further passages.

### Other viral sequences identified in cloacal samples

A total of 11 contigs ranging from 769 to 8768 nucleotides were assembled and identified by MetaViC. Based on BLAST analysis these contigs were classified in six viral families (*Polycipiviridae*, *Iflaviridae*, *Tymoviridae*, *Dicistroviridae*, *Fusaviridae*, and *Nodaviridae*), one contig in Ourmiavirus-like (Unclassified viral family), and another contig remained unclassified (Table 2). All these sequences were related to viral families that infect invertebrates and plants, suggesting that they might be ingested in the birds’ diet.

**Table 2:** Novel viruses possibly from dietary of birds.

GenBank	Virus	Size in nucleotides	Family	Virus related	Amino acid identity (%)	Gene	Pool
MK189082	Breves polycipivirus	1861	*Polycipiviridae*	Solenopsis invicta virus 2^a^	48	RNA-dependent RNA polymerase	02
MK189083	Breves iflavirus	769	*Iflaviridae*	Robinvale bee virus 2^b^	34	Structural polyprotein	04
MK189084	Coroa do aviao tymovirus	1885	*Tymoviridae*	Kennedya yellow mosaic virus^c^	36	Replicase	42
MK189085	Coroa do aviao tymovirus 2	2570	*Tymoviridae*	Citrus sudden death-associated virus^d^	57	RNA-dependent RNA polymerase	42
MK189086	Pernambucos ourmiavirus	2521	Ourmiavirus	Botrytis ourmiavirus^i^	33	RNA-dependent RNA polymerase	44
MK189087	Coroa do aviao dicistrovirus	8768	*Dicistroviridae*	Fesavirus 3^f^	45	Complete coding sequence	44
MK189088	Pernambuco virus	3790	Unclassified	Thika virus^g^	63	Putative polyprotein	44
MK189089	Coroa do aviao iflavirus	9794	*Iflaviridae*	La Jolla virus^e^	51	Putative polyprotein	44
MK189090	Pernambuco mycovirus 1	6472	*Fusaviridae*	Penicillium roqueforti ssRNA mycovirus 1^h^	41	RNA-dependent RNA polymerase	44
MK189091	Pernambuco nodavirus	1077	*Nodaviridae*	Pariacoto virus^j^	41	Protein A	45
MK189092	Pernambuco polycipivirus 2	960	*Polycipiviridae*	Lasius niger virus 1^k^	46	Capsid protein	48

Genbank accession number ^a^MG676340; ^b^MG995719; ^c^NC_001746; ^d^NC_006950; ^e^NC_027128; ^f^MG676340; ^g^NC_027127; ^h^NC_024699; ^i^NC_028476; ^j^NC_003691; ^k^NC_035456.

## Discussion

Birds are a group of vertebrates that include approximately 10,000 species classified within the class *Aves*. They are the natural reservoir of several viral species known to cause significant disease burden in humans and animals, such as influenza viruses, West Nile virus and Newcastle disease virus. Therefore, migratory birds play an important role in the emergence and dissemination of pathogenic viruses^35^. In recent years, extensive metagenomic studies have dramatically expanded our knowledge about the virosphere, including the discovery of novel viruses in domestic and wild bird species^36^. Here, we identified and characterized the genomes of a novel astrovirus and calicivirus species identified in wild birds captured in Brazil.

RtAstV possesses a typical genome organization of astroviruses^1^. However, we observed an overlapping region of 85 nt between ORF1a and ORF1b, which has been described only in an astrovirus detected in the intestines of chickens affected with the runting-stunting syndrome (RSS)^37^. Based on ICTV species demarcation criteria of the *Astroviridae* family, a novel avastrovirus species should share between 33.8 and 78.3% amino acid distance in the complete ORF2 region^4^. Considering the RtAstV shared only 66% amino acid distance, we propose that the RtAstV should constitute a novel species member within the genus *Avastrovirus*. Phylogenetic analysis based on amino acid sequences of ORF1a, ORF1b, and ORF2 showed that RtAstV forms a monophyletic clade with Avian nephritis virus 1 (Genbank No. HM029238) identified in healthy chicken flocks in China^34^, and avian nephritis virus (Genbank No. NC_003790) associated with acute nephritis in chickens worldwide^8^. However, we identified the RtAstV in only one cloacal sample.

Caliciviruses have been detected in an extensive broad range of vertebrate hosts. Here, we described and characterized the first calicivirus identified in migratory birds. RCTV exhibits the typical organization of caliciviruses, encoding a polyprotein and capsid protein^15^. Interestingly, our phylogenetic analysis revealed that RCTV forms a monophyletic clade with caliciviruses identified only in poultry so far, which has been proposed as a genus *Nacovirus*^38^. This putative novel genus is composed by caliciviruses identified in poultry, including chicken, goose, and turkey from Brazil, Germany, and China^19,38–40^. Our RCTV sequence was obtained from a single and apparently healthy bird.

Based on our knowledge of the natural hosts of the astroviruses and caliciviruses^15,41^, we have performed viral isolation attempts using Vero and UMNSAH/DF-1, which are classical for mammal viruses and standard cell line of bird, respectively. Unfortunately, our attempts to isolate both RtAstV and RCTV were unsuccessful. In both cases, we detected viral RNA in inoculated cells after the first blind passage. However, this could be due to the presence of residual RNA from the inoculum. Further studies are needed to investigate the aetiological role and pathogenic potential of the viruses described here.

The novel astrovirus and calicivirus were identified in distinct samples of ruddy turnstone (*Arenaria interpres*), a migratory bird with an extensive geographical distribution worldwide. In the Americas, ruddy turnstones use breeding grounds in the coast and also up to several kilometers inland in Alaska and the Canadian Arctic during June and July^42^. During the boreal winter, ruddy turnstones arrive in Brazil around September via the Western and Eastern Amazon, the Central Plateau and the Atlantic coast. They remain in the country until April^42,43^. So far, influenza, coronaviruses, and avian paramyxoviruses have been identified in ruddy turnstone, including an H11N9 avian influenza virus^43–45^. Therefore, the ruddy turnstone can be a source of viral spreading in the Americas, including both viruses described in this study.

All the viral sequences were detected in cloacal swabs samples and included sequences related to invertebrate and plant viruses that we assumed to be ingested with the birds’ diet as has been described previously^46^.

In sum, we have expanded the viral diversity of migratory birds with the notable characterization of novel astroviruses and calicivirus from ruddy turnstone captured in the northeast coast of Brazil. These findings shed new light on diversity, ecology and host range of the families *Astroviridae* and *Caliciviridae*.

## Materials and Methods

### Bird samples and ethical statements

A total of 118 samples were obtained from eight different birds species were random collected from 2006 to 2010 from three sites in the north and northeast region of Brazil. Poultry samples (*Gallus gallus* and *Cairina moschata*) were collected in Breves municipality, Pará State. Samples obtained from migratory birds (*Arenaria interpres*, *Thalasseus sandvicensis* and *Calidris pusilla*) were collected in Coroa do Avião islet in Pernambuco State, and samples obtained from wild residents birds (*Hylophilus amaurocephalus*, *Sakesphorus cristatus*, and *Coryphospingus pileatus*) were collected in São José do Egito municipality, Pernambuco State (Fig. 1). All captured birds were apparently healthy with no observable signs of disease. Bird species were identified using morphological characteristics keys as previously described^47,48^. All procedures, protocols, and methods performed in this study were approved based on guidelines and regulations of Ethics Committee for Animal Research from University of São Paulo and Chico Mendes Institute for Biodiversity Conservation of Brazilian Ministry of the Environment (No. 25895–1).

### Preparation of samples, genome sequencing and assembly

Individual samples were clustered in fifteen pools based on bird species, sample type (i.e., cloacal swab or sera), date and place of collection (Table 1), and the pools were processed as previously described^49^. To remove naked DNA and RNA, 200 μl of the resuspended pellet from each pool were digested in a cocktail with 20U of Turbo DNase (Life Technologies, USA), 25U of benzonase (Sigma-Aldrich, USA), and 0.1 mg/ml of RNase A (Life Technologies, USA) at 37 °C for 2 hours in 20 μl of 10X DNase buffer (Life Technologies, USA). Subsequently, the viral genomes were extracted with a QIAamp viral RNA mini kit (Qiagen, Hilden, USA). cDNA was synthesized using Superscript II cDNA synthesis kit and random hexamers (Invitrogen, Carlsbad, USA) according to the manufacturer’s instructions. Then, cDNAs were prepared for high-throughput sequencing using TruSeq Universal adapters (Illumina, San Diego, USA) and standard multiplex adaptors. A paired-end, 150-base-read protocol in *RAPID* module was used for sequencing in an Illumina HiSeq 2500 instrument as recommended by the manufacturer. Sequencing was performed in the Life Sciences Core Facility (LaCTAD) at the State University of Campinas (UNICAMP), Brazil. Sequencing reads were *de novo* assembled using the MetaViC pipeline (available on https://github.com/sejmodha/MetaViC) as previously described^50^.

### Viral genome characterization

Viral genome sizes and ORFs were predicted using Geneious 9.1.2 (Biomatters, Auckland, New Zealand) and confirmed using the BLASTX database. Protein domains were screened using InterProScan^51^.

### Phylogenetic analysis

Maximum likelihood (ML) phylogenetic trees were inferred using amino acid sequences of viruses described in this study with representative members of each viral family. Multiple sequence alignments (MSA) were generated using PROMALS3D^52^ with manual adjustments. ML trees were inferred using IQ-TREE version 1.4.3 software using 1,000 ultrafast bootstraps and the best-fit amino acids model determined by Bayesian Information Criterion, which considered 144 reversible amino acids substitution models^53,54^. Statistical support for individual nodes was estimated using the bootstrap value. Phylogenetic trees were visualized using FigTree (v.1.4.2). In addition, MSAs were used in p-distance analyses to calculate the amino acid evolutionary distances among the identified viruses and representative members of the same virus family. All ambiguous positions were removed for each sequence pair. Standard error estimations were calculated by bootstrapping (1,000 replicates) using MEGA (v.7)^55^.

### Recombinant events analysis

To identify potential recombinant events of the novel viruses, MSAs at the nucleotide level were analyzed using the RDP, GENECONV, Bootscan, MaxChi, Chimaera, SiScan and 3Seq methods implemented in the RDP4 program^56^. Default program settings for all methods were used to perceive sequences as linear, to require phylogenetic evidence, to refine breakpoints and to check alignment consistency. The highest acceptable P value was set at 0.05, after considering Bonferroni correction for multiple comparisons. All method-specific program settings remained at their default values.

### RT-PCR for novel astrovirus and calicivirus

To determine the authenticity and frequency of astroviruses and caliciviruses in bird samples identified by HTS, viral RNA of all individual samples was extracted using QIAamp viral RNA extraction kit (Qiagen, Hilden, Germany). Then, all samples were screened by RT-PCRs using primer sets as previously described^33,57^. Amplicons were visualized by gel electrophoresis in 1.5% agarose gels. All PCR products were verified by dideoxy sequencing using ABI 3730 genetic analyzer (Applied Biosystems, Foster City, USA).

### Cells and experimental infections

Vero and UMNSAH/DF-1 cells were propagated as previously described using D-MEM containing 10% fetal bovine serum (FBS), 100 U/ml of penicillin and 100 μg/ml streptomycin at 37 °C with 5% CO_2_ ^58–61^. Samples were filtered through a 0.22-μm filter, and 250 μl was inoculated onto cell monolayers in T25 flasks. Flasks were gently rocked for 1 hour at 37 °C before 7 ml of the respective culture media containing 4% FBS was added. Inoculated cells were incubated for seven days. Supernatants were passaged three times in each cell line, and for each passage, RNA was extracted from cells and supernatant. Virus infection was assessed by RT-PCR and Sanger sequencing, as described above.

## Supplementary information

Supplementary Information

## Data Availability

All sequence reads generated in this project are available under the NCBI Short Read Archive (SRA) under accessions SAMN09843574-SAMN09843583 (BioProject ID: SRP158341) and all consensus virus genome sequences have been deposited in GenBank (accession numbers: MK189082-MK189094).
