# Supplementary material for: Discovery of novel astrovirus and calicivirus identified in ruddy turnstones in Brazil
Source: Sci Rep. 2019 Apr 3;9:5556. doi: 10.1038/s41598-019-42110-3 (PMC6447618; doi:10.1038/s41598-019-42110-3)

Title: Discovery of novel astrovirus and calicivirus identified in ruddy turnstones in Brazil

Running Title: Novel astrovirus and calicivirus in migratory birds

Authors: William Marciel de Souza[1,2,3,¶,*], Marcílio Jorge Fumagalli[1,¶], Jansen de Araujo[4], Tatiana Ometto[4], Sejal Modha[2], Luciano Matsumiya Thomazelli[4], Edison Luís Durigon[4], Pablo Ramiro Murcia[2] and Luiz Tadeu Moraes Figueiredo[1].

Affiliations:

[1]Virology Research Center, School of Medicine of Ribeirão Preto of University of São Paulo, Ribeirão Preto, 14049-900, SP, Brazil.

[2]MRC-University of Glasgow Centre for Virus Research, Glasgow, G61 1QH, Scotland, United Kingdom.

[3] Department of Genetics, Evolution and Bioagents, Institute of Biology, University of Campinas, 13083-862, Campinas, São Paulo, Brazil.

[4] Institute of Biomedical Sciences, University of São Paulo, São Paulo, 05508-900, SP, Brazil.

[¶] These authors contributed equally to this work.

***Corresponding author:** wmarciel@usp.br or wmarciel@hotmail.com.

Supplementary Table and Figures

Supplementary Table 1. Distribution of reads assembled by MetaViC per organism.

ID	Eukaryote	Bacteria	Viruses	Archaea	Unassigned
1	47% (2136)	52% (2381)	0.5% (22)	0.4% (20)	0.3% (14)
2	44% (210)	43% (202)	10% (46)	-	1% (6)
3	64% (109)	29% (49)	5% (9)	-	1% (2)
4	68% (102)	23% (34)	9% (13)	-	-
40	53% (85)	28% (44)	17%(27)	-	-
41	51% (201)	43% (171)	3% (13)	-	1% (5)
42	52% (98)	30% (57)	16% (30)	-	1% (2)
43	55% (68)	33% (41)	9 % (11)	-	-
44	53% (1372)	42% (1070)	5% (125)	-	-
45	80% (1071)	17% (228)	2% (25)	-	0.5% (6)
46	70% (234)	26% (86)	3% (9)	-	-
47	79% (4724)	21% (1243)	0.5% (19)	-	-
48	48% (756)	47% (739)	2% (28)	-	-
49	64% (61)	29% (28)	7% (7)	-	-

Numbers of reads are showed between parentheses.

Supplementary Figure 1. Mapping of reads against genomes of Ruddy turnstone astrovirus.

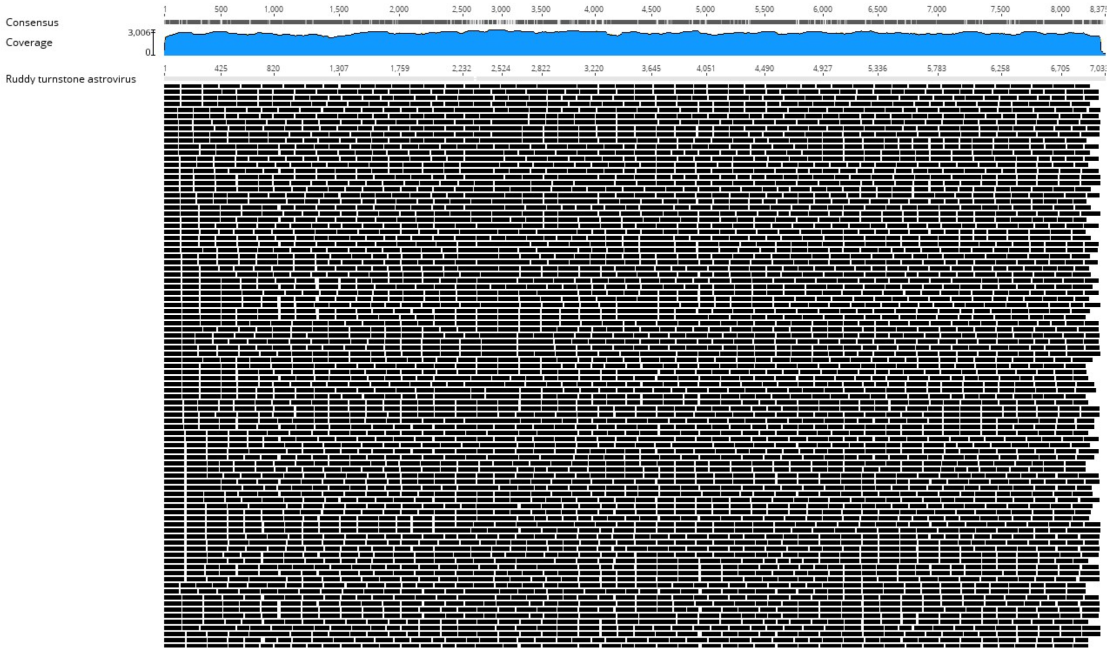

Supplementary Figure 2. Mapping of reads against genomes of Ruddy turnstone calicivirus.

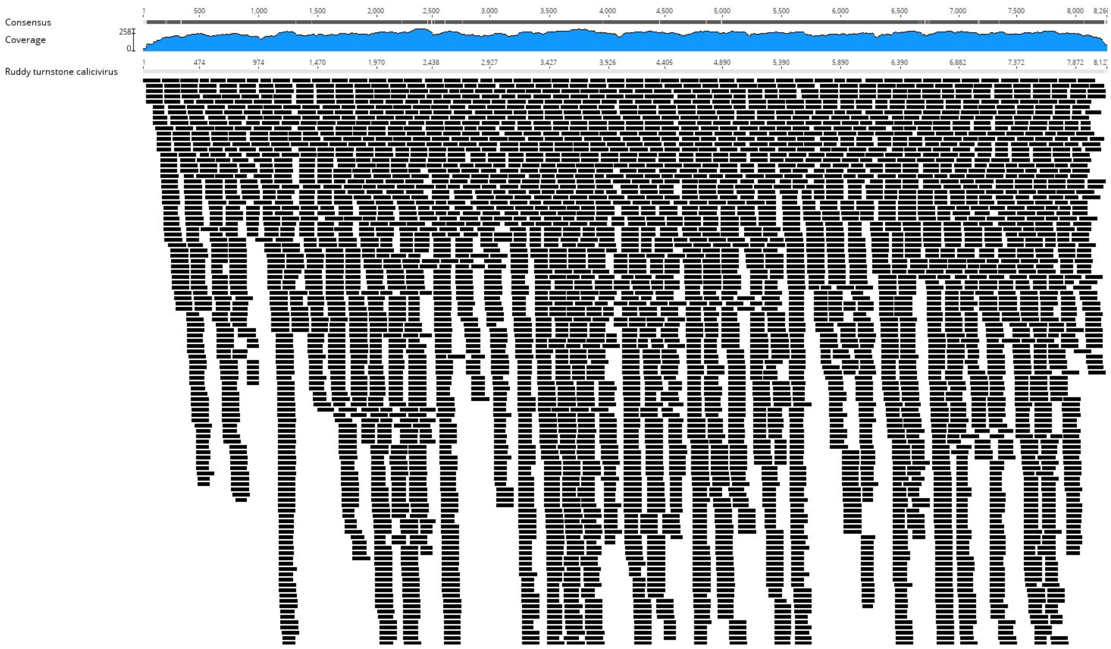

Supplement: Supplementary file 1 — Supplementary Information [file 41598_2019_42110_MOESM1_ESM.pdf]
